# Detection of tumor-associated cells in cryopreserved peripheral blood mononuclear cell samples for retrospective analysis

**DOI:** 10.1186/s12967-016-0953-2

**Published:** 2016-07-02

**Authors:** Peixuan Zhu, Melissa L. Stanton, Erik P. Castle, Richard W. Joseph, Daniel L. Adams, Shuhong Li, Platte Amstutz, Cha-Mei Tang, Thai H. Ho

**Affiliations:** Creatv MicroTech, Inc., 11609 Lake Potomac Drive, Potomac, MD 20854 USA; Department of Laboratory Medicine and Pathology, Mayo Clinic, 13400 East Shea Blvd, Scottsdale, AZ 85259 USA; Department of Urology, Mayo Clinic Hospital, 5777 E Mayo Blvd, Phoenix, AZ 85054 USA; Division of Hematology and Medical Oncology, Mayo Clinic, 4500 San Pablo Rd, Jacksonville, FL 32224 USA; Division of Hematology and Medical Oncology, Mayo Clinic, 13400 East Shea Blvd, Scottsdale, AZ 85259 USA

**Keywords:** Circulating cancer-associated macrophage-like cells (CAMLs), CellSieve™ microfiltration, Cryopreservation, Circulating tumor cells (CTCs), Ficoll, Fluorescence antibody staining

## Abstract

**Background:**

Cryopreserved peripheral blood mononuclear cells (PBMCs) are commonly collected in biobanks. However, little data exist regarding the preservation of tumor-associated cells in cryopreserved collections. The objective of this study was to determine the feasibility of using the CellSieve™ microfiltration assay for the isolation of circulating tumor cells (CTCs) and circulating cancer-associated macrophage-like cells (CAMLs) from cryopreserved PBMC samples.

**Methods:**

Blood samples spiked with breast (MCF-7), prostate (PC-3), and renal (786-O) cancer cell lines were used to establish analytical accuracy, efficiency, and reproducibility after cryopreservation. The spiked samples were processed through Ficoll separation, and cryopreservation was followed by thawing and microfiltration.

**Results:**

MCF-7 cells were successfully retrieved with recovery efficiencies of 90.5 % without cryopreservation and 87.8 and 89.0 %, respectively, on day 7 and day 66 following cryopreservation. The corresponding recovery efficiencies of PC-3 cells were 83.3 % without cryopreservation and 85.3 and 84.7 %, respectively, after cryopreservation. Recovery efficiencies of 786-O cells were 92.7 % without cryopreservation, and 82.7 and 81.3 %, respectively, after cryopreservation. The recovered cells retained the morphologic characteristics and immunohistochemical markers that had been observed before freezing. The protocols were further validated by quantitation of CAMLs in blood samples from two patients with renal cell carcinoma (RCC). The recovery rates of CTCs and CAMLs from cryopreserved samples were not statistically significant different (P > 0.05) from matched fresh samples.

**Conclusions:**

To our knowledge, this is the first report that CAMLs could be cryopreserved and analyzed after thawing with microfiltration technology. The application of microfiltration technology to cryopreserved samples will enable much greater retrospective study of cancer patients in relation to long-term outcomes.

**Electronic supplementary material:**

The online version of this article (doi:10.1186/s12967-016-0953-2) contains supplementary material, which is available to authorized users.

## Background

Circulating tumor-associated cellular structures, such as circulating tumor cells (CTCs) and circulating cancer-associated macrophage-like cells (CAMLs) in blood, have emerged as important prognostic and predictive biomarkers for diagnosis, prognosis, and management of cancer diseases [1–6]. A blood based biopsy has advantages over traditional tissue biopsy, including lower cost, reduced risk, and repeatability. These assays are usually performed on fresh blood samples within a limited timeframe after blood draw. Little is known about the effects of cryopreservation on the recovery and characterization of all types of tumor-associated cells. Furthermore, assays compatible only with fresh samples require clinical on-site processing, because many factors, including sample transportation, age of blood, temperature fluctuations, and preservative reagents, can affect assay performance and reproducibility.

Prospective cryopreservation is a common method for long-term storage of biospecimens and is an invaluable tool for retrospective clinical studies of selected populations [7–9]. However, these biospecimens may be incompatible with assays that require intact cell membranes, because of ice crystal formation and subsequent lysis during the thawing process. Thus, assays compatible with current cryopreservation methods would allow the retrospective analysis of biomarkers in patient cohorts with known clinical outcomes. Additional advantages include (1) performing the assay in batches in a centralized laboratory to minimize interlaboratory variations and (2) assaying longitudinal collections together to minimize interassay variations.

Microfiltration is an effective method for isolating CTCs and other cells of interest from several types of cancers [1–3, 10–14]. CellSieve™ microfiltration technology, developed by Creatv MicroTech, Inc, uses a novel filter membrane with a high density of evenly distributed pores (160,000 pores per filter) with precisely controlled pore size (7 µm) to achieve high capture efficiency of tumor cells and low contamination of blood cells. The membrane material has low background fluorescence that allows the cells to be further characterized through various fluorescence-based assays.

The objective of our study was to determine whether CellSieve™ microfiltration technology can be applied to recovery and analysis of CTCs and CAMLs from cryopreserved PBMC samples. Initially, we used blood samples spiked with three cancer cell lines, MCF-7, PC-3, and 786-O, to demonstrate technical feasibility of the system. The protocols were validated by analyzing CAMLs recovered from cryopreserved PBMC samples of patients with renal cell carcinoma (RCC).

## Methods

### Cell lines and reagents

786-O (renal cell carcinoma), MCF7 (breast cancer), PC-3 (prostate cancer), SKBR3 (breast cancer), MDA-MB-231 (breast cancer), LNCaP (prostate cancer), and primary HUVEC (human umbilical vein endothelial cells) cell lines and others as previously described [1, 2, 15] were all obtained from American Type Culture Collection (ATCC). The cell lines were cultured using conditions specified by ATCC protocols. Cell suspensions were prepared with standard trypsin-treatment methods. Total cell counts were determined manually with a hemocytometer. Cell viability was assessed with trypan blue dye exclusion assay. Fresh cells with viability greater than 98 % were used for spiking experiments. CellSieve™ CTC Enumeration Kits (Creatv MicroTech, Inc) were used for recovery and fluorescence antibody staining of the filter-captured cells. These kits contain CellSieve™ filter membranes and reagents required for microfiltration of blood and/or PBMC samples and characterization of the filter-captured cells, including ready-to-use prefixation buffer, postfixation buffer, permeabilization buffer and mounting solution with DAPI (4′,6-diamidino-2-phenylindole), and antibody cocktail mixtures at optimized concentrations. To stain breast or prostate cancer cells, antibody mixture containing cytokeratin (CK) 8, 18, 19/fluorescein (FITC), epithelial cell adhesion molecule (EpCAM)/phycoerythrin (PE), CD45/Cyanine 5 was used. To stain RCC cells, antibody mixture containing CK8, 18, 19/FITC, vimentin/eFluor^®^ 615 (EF615) and CD45/Cyanine 5 was used.

### Patient eligibility and recruitment

Subjects eligible for enrollment in the Multidisciplinary Genitourinary Diseases Biospecimen Bank were those seen at Mayo Clinic’s campus in Scottsdale, Arizona, and were ≥18 years of age, able to provide informed consent, and undergoing evaluation to determine eligibility as a kidney donor or for treatment of genitourinary diseases. During the course of routine clinical visits at Mayo Clinic, subjects were consented to participate in the Biospecimen Bank. The protocol for collecting biospecimens was approved under a Mayo Clinic Institutional Review Board (IRB) approved protocol (08-000980). For healthy control subjects, blood samples were collected at Creatv MicroTech from healthy donors signed informed consent under a protocol approved by the Western IRB.

### CellSieve™ microfiltration platform

**Fig. 1:**
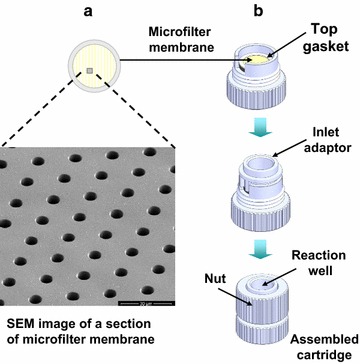
Schematic representation of assembly and setup of the CellSieve™ Microfiltration System. **a** Microfilter membrane. **b** Assembly of the filter cartridge

The CellSieve™ microfiltration platform has been developed by Creatv MicroTech [1, 2, 15]. This platform consists of three basic components: a filter membrane and cartridge (Creatv MicroTech, Inc), and a programmable syringe pump system (KD Scientific). The filter membrane has a 9-mm diameter filtration area containing a high density of pores (approximately 160,000 pore density and 7-µm pore size). Figure 1a shows a scanning electron microscope (SEM) image of the filter and Fig. 1b illustrates a schematic diagram of assembling the microfilter into the filter cartridge. The assembled cartridge is connected to an input syringe with an inlet fitter to introduce the blood sample and a waste syringe to generate negative pressure. This assembly was installed on the syringe pump. The syringe pump is programed to pull to deliver a selected flow rate of blood through the microfilter. After filtration and washes, the syringe barrel and inlet fitting are removed. The remaining parts of the cartridge are used as a reaction chamber, allowing for performing assay steps directly within the chamber. Reagents were added into the chamber with pipetting and drawn into the waste syringe by the syringe pump.

Compared with conventional microfiltration methods, the CellSieve™ microfilter membrane provides (i) precise uniform pore size and distribution for maximal filtration efficiency, (ii) high porosity for fast filtration process, (iii) low background allowing high quality images, (iv) flat surface on the microscope slide minimizing the need for refocusing during microscopy, and (v) strong material to avoid breakage. To eliminate unnecessary handling procedures, such as filter disassembly for immunofluorescence, a filter cartridge is designed to aid in processing sample microfiltration followed by on-cartridge assay procedures; all of the assay procedures including antibody staining can be completed within the cartridge. With multiple cartridges, multiple blood samples can be analyzed simultaneously. Furthermore, the use of a programmable syringe pump provides consistent flow rate. The overall sample processing time is less than 2.5 h.

### Microfiltration of healthy blood samples spiked with cell lines

Samples of whole blood were collected from healthy donors into sodium heparin Vacutainer tubes (Becton, Dickinson and Co). Separation of PBMC was performed with Ficoll-Paque (GE Healthcare) according to the protocols used in Mayo Clinic’s biobank for processing clinical samples [8]. 7.5 mL of whole blood was centrifuged at 1600 rpm for 15 min. Plasma was removed and replaced with the same volume of phosphate buffered saline (PBS). A cell suspension was prepared by 1:1 diluted the cell sample with PBS. Ten mL of Ficoll-Paque solution (GE-Healthcare) was added in a 50 mL conical tube. The diluted cell suspension was then slowly and carefully layered on the top of Ficoll-Paque solution without mixing. The sample was centrifuged at 400×*g* for 30 min at 20 °C in a swinging bucket rotor with brake-off. Immediately after centrifugation, the upper layer was aspirated and discarded. The mononuclear cell layer was carefully transferred to a new 15-mL conical tube and mixed with PBS to a total of 15 mL. The sample was centrifuged at 300×*g* at room temperature for 10 min with brake-on. The supernatant was removed and discarded. The PBMC was gently washed one more time with PBS. The cells pellet was resuspended in 1 mL of cryogenic medium (10 % dimethyl sulfoxide and 90 % fetal bovine serum), and transferred into a Nunc (Sigma-Aldrich Co LLC) cryovial. The tubes were placed on Mr. Frosty tube racks and immediately placed in dry ice (less than 10 min at room temperature). The cryovials on the rack were directly stored at −80 °C freezer overnight. The frozen cryovials were transferred and placed in liquid nitrogen if it was needed.

For spiking experiments, a defined number of live tumor cells were spiked into the blood samples. The spiked samples were subjected to Ficoll separation to isolate fractions containing PBMCs and tumor cells. The mononuclear cells were washed, suspended in 1 mL of cryogenic medium, and transferred into a Nunc cryovials (1 mL/vial). For each cell line, four vials of sample were prepared. One vial was used as a control, with no cryopreservation. The other three vials were stored at −80 °C until thawed. After a period of storage, the frozen samples were quickly thawed in a 37 °C water bath, followed by immediate processing through CellSieve™ microfiltration in accordance with Creatv MicroTech’s protocol. Briefly, the filter membrane was rinsed with 5 mL of PBS. Each of the cell samples was washed, prefixed, and then filtered through the filter membrane (flow rate, 5 mL/min). The filter membrane was washed five times with PBS. The cells on the membrane were further treated with postfixation and permeabilization buffers (Creatv MicroTech, Inc.).

To stain recovered MCF-7 and PC-3 cells, we added 150 µL of fluorescent antibody mixture, against cytokeratins 8, 18, 19/FITC, EpCAM/PE, and CD45/Cyanine5, and incubated the samples at room temperature for 1 h. To stain recovered 786-O cells, fluorescent antibody mixture against cytokeratins 8, 18, 19/FITC, Vimentin/EF615, and CD45/Cyanine5 was used. Unbound antibodies were washed away with PBS.

After completion of the assay, the filter cartridge was disassembled and the filter membrane removed and placed onto a clean microscope slide, then mounted with 10 µL of mounting solution with DAPI and a cover slip for microscopy examination. The positivity of each marker was defined for any cells with a relative fluorescence signal greater than threefold over the background. The majority of cells were the single cells. For a cluster of small number of cells that were aggregated into a clump, we counted each cluster as one cell. The recovered tumor cells were counted from five fields of view under the 10× objective covering about 5 % of the filter area, and an average was calculated for estimating the total number of cells on the entire filter membrane.

### Microfiltration of blood samples collected from patients affected by metastatic renal cell carcinoma

Whole blood samples, with three matched tubes for each blood draw, were collected in CellSave tubes (Janssen Diagnostics, LLC) from patients with RCC at Mayo Clinic and shipped to Creatv MicroTech for analysis. The concordance of the recovery was determined through processing of the matched tubes as with cryopreservation and without cryopreservation, respectively. Two of the tubes labeled as without cryopreservation (Tube 1 and Tube 2) were processed through CellSieve™ microfiltration within 24 h after blood draw. The third tube was processed through Ficoll separation, cryopreservation at −80 °C for 7 days, followed by thawing and CellSieve™ microfiltration, and antibody staining. The procedures of microfiltration and antibody staining were the same as used in the spiking experiments. The filter-captured cells from the Tubes 1 and 3 were stained with an antibody mixture consists of fluorescent dye-conjugated antibodies against CK8, 18, 19/FITC, vimentin/EF615 and CD45/Cyanine5. The filter-captured cells from the Tube 2 were stained with an antibody mixture consists of fluorescence-conjugated antibodies against PD-L1/Alexa Fluor 488, vimentin/EF615 and CD45/Cyanine5. The CTC was defined as a nucleated cell with positive staining for CKs and vimentin but negative staining for CD45 for Tubes 1 and 3. The CAML was defined according to criteria described previously [2].

## Results

### Recovery of spiked cell lines in cryopreserved human PBMC samples

To determine whether tumor cells could be isolated from cryopreserved biospecimens, whole blood cell samples from healthy donors were spiked with a defined number of three tumor cell lines (MCF-7, PC-3, and 786-O). The fractions of PBMCs and tumor cells were isolated by Ficoll separation and after thawing, processed with CellSieve™ microfiltration at different time points. To evaluate stability of tumor cells under frozen conditions and whether freeze/thaw procedures affect recovery of tumor cells, we selected 3 time points: day 0, day 7, and day 66. The day 0 sample (fresh) was processed through microfiltration immediately without cryopreservation. The day 7 and day 66 samples were cryopreserved at −80 °C for 7 and 66 days and then thawed for the microfiltration assay. The recovered MCF-7 and PC-3 cells were identified by expression patterns of CKs+/EpCAM+/CD45−, whereas the recovered 786-O cells were identified by CKs+/vimentin+/CD45−. The results of recovery efficiencies are summarized in Table 1.

**Table 1:** Recovery efficiency of spiked tumor cell lines in cryopreserved human PBMC samples

Cell line	Input cells	Day 0^a^		Day 7^b^		Day 66		Mean (SD) %
		Cells recovered, no.	Recovery, %	Cells recovered, no.	Recovery, %	Cells recovered, no.	Recovery, %	
MCF-7	4000	3620	90.5	3510	87.8	3560	89.0	89.1 (1.4)
PC-3	3000	2500	83.3	2560	85.3	2540	84.7	84.4 (1.0)
786-O	3000	2780	92.7	2480	82.7	2440	81.3	85.6 (6.2)

^a^Day 0, before freezing

^b^Day 7 and day 66, after freezing

We obtained mean (standard deviation, SD) spike-in recovery rates of 89.1 (1.0) % for MCF-7, 84.4 (1.4) % for PC-3, and 85.6 (6.2) % for 786-O, respectively. Between the fresh and stored frozen samples, we observed that the cryopreservation/thaw procedure had no significant effect on the recovery efficiencies of MCF-7, PC-3 or 786-O cells (P > 0.05 by 2-way analysis of variance). A 10 % decrease in recovery rates of 786-O was observed from the frozen samples (Day 7, 82.7 %; Day 66, 81.3 %) compared with the fresh sample (Day 0, 92.7 %). The cell line results suggest that the cryopreservation procedure preserves CTCs for microfiltration with minimal loss.

To determine the lowest number of tumor cells that can be detected by the microfiltration system, a series of spiking experiments were performed using six representative cell lines (786-O, MCF7, PC-3, SKBR3, MDA-MB-231, LNCaP). In our spiking experiments, the lowest absolute number of cells detected in our system was 3, 9, 5, 5, 4, and 4 cell using 786-O, MCF7, PC3, SKBR3, MDA-MB231, and LNCaP cell lines, respectively.

The linearity and limit of tumor cell detection in the spiking experiments are shown in Additional file 1: Figure S1. To also determine if cells lacking genome wide chromosomal rearrangements or aneuploidy can be detected by the microfilitration system, we used primary HUVECs for spiking experiments. Similar to MCF-7, PC-3 and 786-O, our observed capture efficiency for HUVECs was greater than 94 % (Additional file 2: Table S1). Microfiltration did not impact the expression of endothelial markers (Additional file 3: Figure S2). We also investigated the effect of storage temperatures at −80 °C and in liquid nitrogen on PBMC samples. No significant differences in cell morphology and antibody staining patterns were observed between cells cryopreserved (at −80 °C) and in liquid nitrogen for three weeks (Additional file 4: Figure S3).

### Expression of epithelial and mesenchymal biomarkers in processed cryopreserved tumor cell lines

**Fig. 2:**
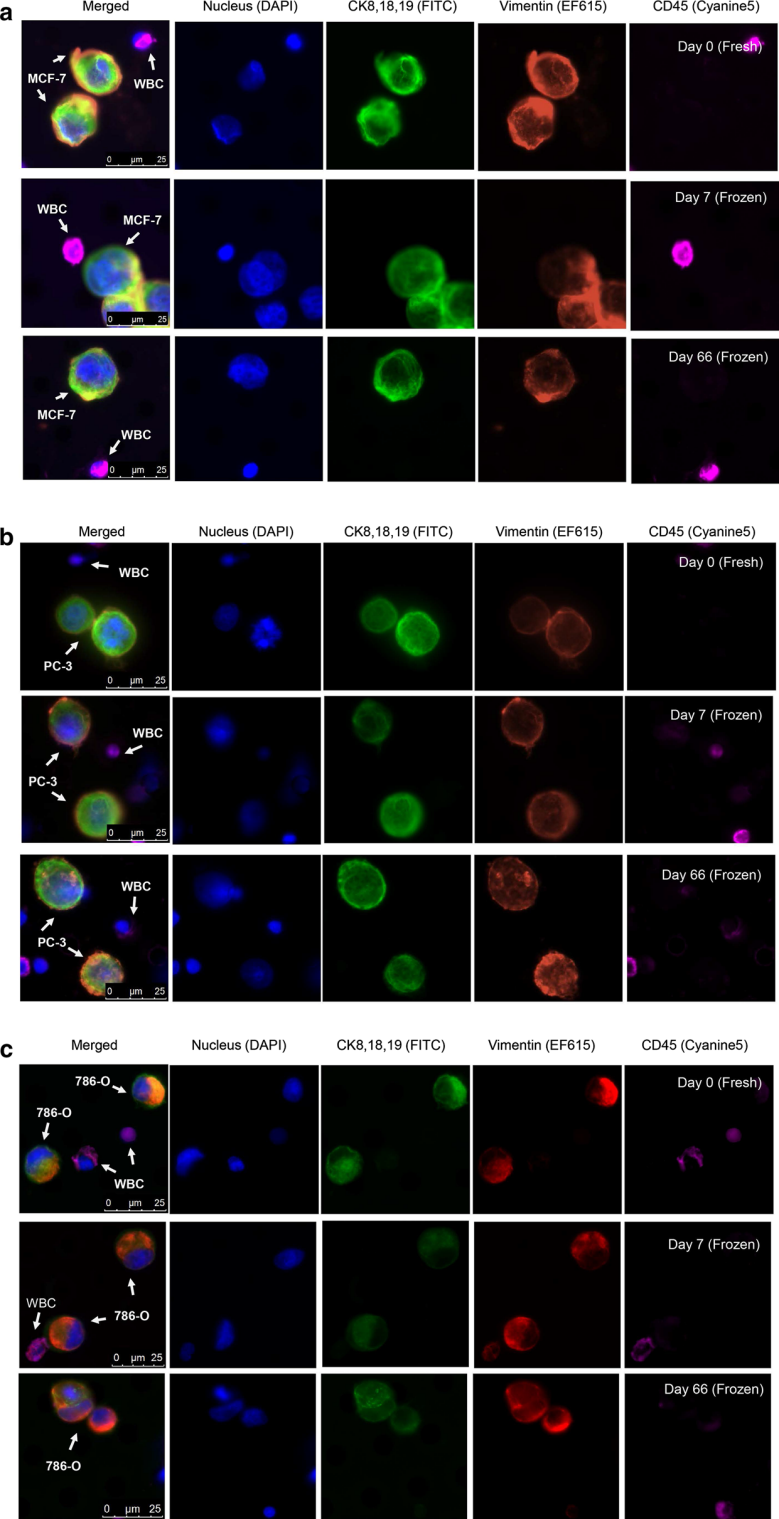
Recovery of tumor cell line cells in cryopreserved peripheral blood mononuclear cells (PBMCs) samples. Healthy blood samples were spiked with three tumor cell lines: MCF-7 (**a**), PC-3 (**b**), and 786-O (**c**). The PBMCs and tumor cells were isolated with Ficoll separation and then cryopreserved. The tumor cells were retrieved after thawing through CellSieve™ microfiltration. Day 0 (fresh) was a control sample without cryopreservation. Day 7 and day 66 (frozen) were the samples after cryopreservation. Each biomarker expression is indicated as CK8, 18, 19/FITC (*green*), EpCAM/PE (*orange*), vimentin/EF615 (*red*), and CD45/Cyanine5 (*magenta*). **a** The MCF-7 and PC-3 cells were stained as a pattern of CK+/EpCAM+/CD45−. **b** White blood cells (WBCs) were stained as CK−/EpCAM−/CD45+. **c** The 786-O cells were stained as CK+/vimentin+/CD45−, whereas WBCs were stained as CK−/vimentin ±/CD45+ because of cross-reactivity of vimentin antibody. The morphologic characteristics and antibody staining patterns of the tumor cells were unchanged after cryopreservation for 66 days

To determine if cryopreservation alters tumor cell lines, the concordance of tumor cell morphologies, as well as antibody staining patterns of the epithelial and mesenchymal markers, was compared among the samples that were processed with or without cryopreservation. No damage, as evidenced by loss of markers, was observed in the recovered cells. The recovered cells retained the morphologic characteristics and markers that were observed before freezing (Fig. 2). The signal and staining patterns of cytokeratins, EpCAM, and vimentin were largely unchanged after cryopreservation. We did not observe any notable differences in staining patterns and staining intensity among the fresh (day 0) specimens vs the frozen specimens (day 7 and day 66). These results confirm that the cytokeratin, EpCAM, and vimentin markers, as well as cell morphologic characteristics, are preserved in the cryopreserved tumor cells.

### Recovery and enumeration of CAMLs in RCC patient samples processed with and without cryopreservation

**Fig. 3:**
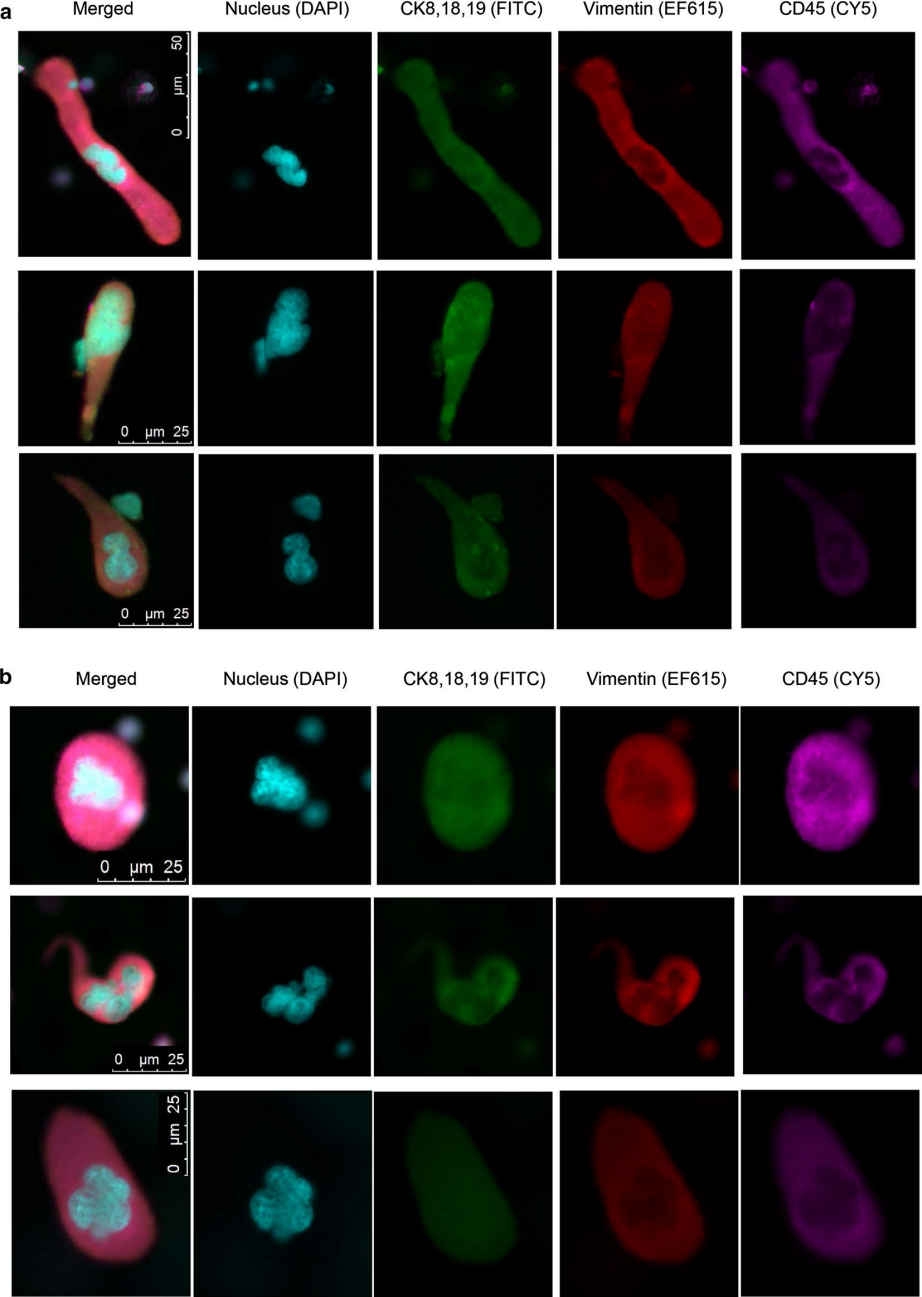
Representative images of cancer-associated macrophage-like cells (CAMLs) detected in the matched renal cell carcinoma samples (RCC patient 1) with or without cryopreservation. Whole blood samples were drawn at three time points and subjected to Ficoll separation. The matched peripheral blood mononuclear cell samples were processed immediately without cryopreservation (fresh) or processed after cryopreservation for 7 days (frozen). The filter-captured cells were stained with CK8, 18, 19/FITC (*green*), vimentin/EF615 (*red*), and CD45/Cyanine5 (*magenta*). Three CAMLs, representing different intensities, are presented for the fresh and frozen samples, respectively. **a** Representative images of typical CAMLs detected in the fresh samples. **b** Representative images of typical CAMLs detected in the frozen samples

In a prior study, we identified CAMLs in peripheral blood from patients with various solid tumor cancers [2]. To determine whether detection of CAMLs is compatible with cryopreservation, three blood samples were collected from a patient affected by stage IV RCC with sarcomatoid differentiation with the primary kidney cancer in place and soft tissue metastases (RCC Patient 1). This patient was selected for study because the samples were known to contain CAMLs defined using criteria from our prior study [2]. Blood samples were collected at three different time points (days 0, 40, and 60). Prior to systemic therapy, the CAML enumeration was highest at 13 per tube. After initiation of systemic therapy, the CAML enumeration decreased to two per tube. At the time of progression on systemic therapy and prior to death, the CAML enumeration increased to 4 per tube. Three matched blood tubes were drawn at defined time points during the treatment course. Tube 1 and Tube 2 were processed through the standard microfiltration procedures without cryopreservation (fresh), whereas Tube 3 was processed through Ficoll separation, cryopreservation, thaw, and microfiltration (frozen). Typical CAMLs were detected in both samples without cryopreservation (fresh) and the matched sample after cryopreservation (frozen) (Table 2; Fig. 3).

**Table 2:** Comparison of RCC patient blood samples processed with or without cryopreservation

Time point	Day	Typical CAML, cells/tube^a^				Denucleated CAML, cells/tube			
		Fresh			Frozen	Fresh			Frozen
		Tube 1	Tube 2	Mean	Tube 3	Tube 1	Tube 2	Mean	Tube 3
1	0	14	12	13	13	4	4	4	5
2	40	1	2	1.5	2	2	3	2.5	2
3	60^b^	3	5	4	4	3	4	3.5	3

*CAML* cancer-associated macrophage-like cell; *RCC* renal cell carcinoma

^a^7.5 mL of whole blood was processed per tube

^b^RCC patient 1 died on day 73

We did not detect statistically significant differences in CAML enumeration among the fresh vs frozen specimens (P > 0.05 by 2-way analysis of variance). We also measured the signal intensities of CKs in the cells from the Tube 1 and Tube 3. The Tube 2 was not used for analysis of signal intensity because it was not stained with CKs. All of the images for CKs were taken under a consistent exposure time (600 ms). Three cytoplasmic positions were selected for measurement of signal intensities of CKs in each CAML, representing the high, majority, and low signals of CKs. The CK signals were plotted versus each CAML cells (Additional file 5: Figure S4). The high and low signals of CKs were indicated as error bars. The background signal of the filter membrane, shown as a dotted line in the plot, was an average signal measured from the fourteen locations on the filter membrane. At Time Point 1, the majority of CK signal intensities in the fresh CAMLs (in range of 460–850, average 616.43 ± 97.72) were similar to that in the frozen CAMLs (range of 522–886, average 619.00 ± 100.71), suggesting that the CKs were preserved after the cryopreservation. At Time Points 2 and 3, the CK signals in the fresh CAMLs were slightly higher than that in the frozen CAMLs. We did not measure the signals of vimentin because different exposure times were used in the imaging to avoid signal saturation.

### Denucleated CAMLs observed in both fresh and frozen RCC samples

**Fig. 4:**
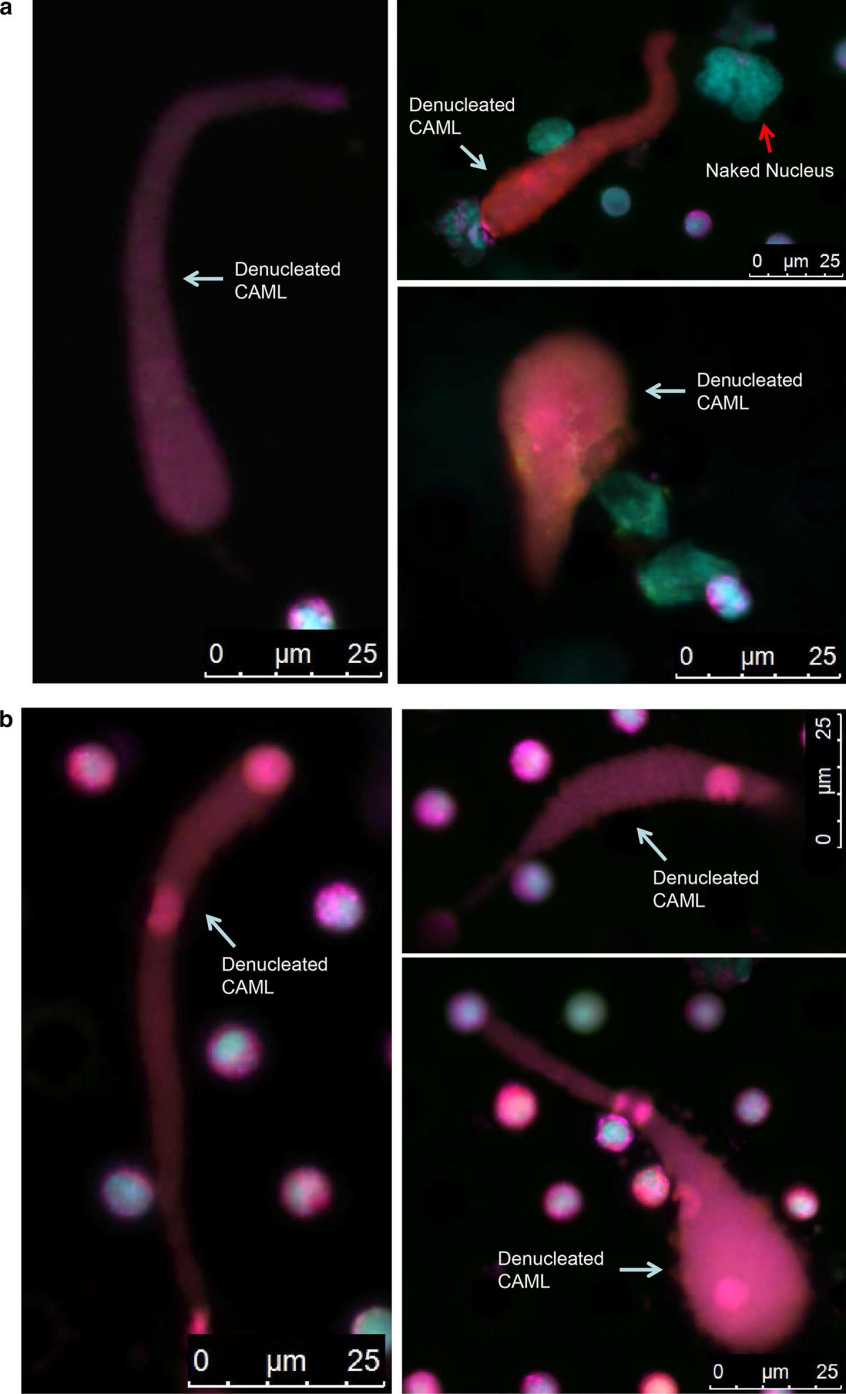
Denucleated cancer-associated macrophage-like cells (CAMLs) detected in the fresh and frozen renal cell carcinoma samples (RCC patient 1). **a** Fresh sample without cryopreservation. A naked multinuclear nucleus, which might be dissociated from the original CAML, is indicated with a *red arrow*. **b** Frozen sample after cryopreservation for 7 days. To better show structural details in nuclei, we used a *light-blue color* for DAPI fluorescence channel. The *small round cells* are white blood cells; the *round-shaped structures* within the denucleated CAMLs may represent white blood cells that were adhered to the filter pores and overlapped with the denucleated CAMLs

In both fresh and frozen samples (RCC Patient 1), we found a cellular structure resembling a typical CAML in morphologic characteristics and antibody staining, but lacking a cell nucleus (Fig. 4; Table 2). This type of cellular structure is designated as *denucleatedCAML* since it has not been described in the literature. The dimension of these denucleated CAMLs varies from 15 to 25 µm wide and 35 to 80 µm long. They show morphologic diversity, with a long shape and sometimes an elongated tail (Fig. 4). We observed a naked, multinuclear nucleus close to the denucleated CAML (Fig. 4a), suggesting that these atypical cellular fragments may result from a previous event of cell dissociation. Furthermore, denucleated CAMLs were also recovered from the cryopreserved samples (Fig. 4b), showing that both intact and denucleated CAMLs were well preserved by cryopreservation.

### Recovery of CTCs in RCC samples processed with and without cryopreservation

**Fig. 5:**
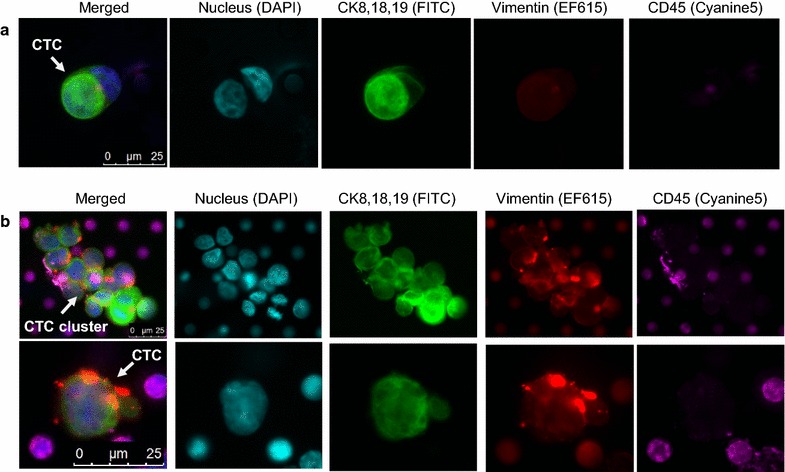
Detection of Circulating tumor cells (CTCs) in the matched fresh and frozen renal cell carcinoma samples (RCC patient 2). A CTC is defined as a nucleated cell positive for both CKs (*green*) and vimentin (*red*) but negative for CD45 (*magenta*). **a** Fresh peripheral blood mononuclear cell sample from a selected patient with renal cell carcinoma, without cryopreservation. **b** Frozen peripheral blood mononuclear cell sample from the same patient with renal cell carcinoma, with cryopreservation for 7 days. The *top row* shows a cluster of CTCs with one cell in mitosis

CTCs have been characterized in the peripheral blood of patients with metastatic RCC. To determine whether CTCs from RCC patients could be preserved with cryopreservation, we identified another stage IV RCC patient with clear cell histology and prior nephrectomy with bone metastases on systemic therapy (RCC Patient 2). In this patient, we identified typical cytokeratin-positive CTCs. The CTC was defined as a cell with a large tumor-like nucleus that stained positive to cytokeratin and vimentin and negative to CD45. One individual CTC was detected in the fresh blood sample from this selected patient. In parallel, one individual CTC plus one CTC cluster were detected in the matched frozen sample. Our results demonstrated that those CTCs organized as a cluster are preserved after Ficoll separation, cryopreservation, thawing, and CellSieve™ microfiltration. The CTCs of the fresh sample expressed high levels of cytokeratin with typical intracellular network of filament structure, which was also observed in the CTCs from the cryopreserved sample (Fig. 5). The anti-vimentin antibody showed cross-reactivity with some white blood cells, but the anti-cytokeratin antibodies were more specific for tumor cells with less cross-reactivity with white blood cells. Moreover, we observed mitotic figures in the CTC cluster. The chromosomes in one CTC nuclei were separated into two identical sets, each in its daughter nucleus (Additional file 6: Figure S5). Our data suggest that our cryopreservation technique is compatible with preserving mitotic events, CTC morphologic characteristics, and CTC biomarkers.

## Discussion

Analysis of tumor-associated cells in peripheral blood offers the advantage of a noninvasive approach to study changes in biomarkers associated with chemotherapy, tumor recurrence, or tumor treatment. However, many CTC technologies require the processing of fresh blood samples and are incompatible with cryopreserved samples, limiting the ability to perform retrospective studies on archived samples. This limitation may pose a challenge in multicenter clinical trials and require a central laboratory to process samples on the same day for the CTC assays. The assay reproducibility may be adversely affected by performing each fresh blood sample individually or by transportation of specimens between locations. Cryopreservation is one of the most commonly used methods for storage biologic specimens collected over time.

We optimized a cryopreservation work flow compatible with detecting tumor-associated cells in stored peripheral blood to overcome assay limitations that require processing of fresh blood. We showed the feasibility of CellSieve™ technology for isolation and identification of CTCs and CAMLs from cryopreserved samples. Our notable observations include (1) recovery of spiked tumor cells lines in cryopreserved peripheral blood using the CellSieve™ platform; (2) preservation of cellular morphologic characteristics and markers in cryopreserved samples from patients with metastatic RCC; and (3) preservation of CTC and CAML enumerations in samples cryopreserved up to 66 days at −80 °C.

When evaluating our observations, we found limitations to the present investigation. First, we analyzed 1 CTC microfiltration platform, and the cryopreservation procedure may not be compatible with other antigen-dependent or antigen-independent cell capture methods. Second, although we evaluated breast, prostate, and kidney cancer cell lines, we analyzed CTCs and CAMLs only in samples from patients with kidney cancer. The DMSO concentration or Ficoll separation may not be compatible with all types of cancer diseases, and further study is required to determine whether the cryopreservation procedure is suitable for other cancers. Third, we did not extend the study beyond 60 days, and the impact of storage beyond this time point may or may not have effect on enumeration assays.

Cryopreservation of PBMCs allows phenotypical and genetic assays to be performed in batches at a central laboratory and minimize assay variation. We have demonstrated that the CellSieve™ system is compatible with Ficoll separation and cryopreservation for detection of CTCs and CAMLs. Investigators have previously reported that CTCs can be recovered from cryopreserved PBMC samples [7, 9, 16]; however, it was unclear whether CAMLs can be recovered from cryopreserved PBMC sample. To our knowledge, we are the first to report that CAMLs can be separated by Ficoll method, cryopreserved for future use, and retrieved after thawing samples stored up to 60 days at −80 °C. In our Ficoll separation, CAMLs segregate to the interphase between Ficoll and plasma, where PBMCs and CTCs are located. CAMLs may have similar density to other mononuclear cells, such as lymphocytes and CTCs, allowing them to be enriched through Ficoll separation, and use of a microfiltration system can isolate cells on the basis of pore size. These enriched cells are suspended in cryoprotectant medium, which contains DMSO to reduce cell lysis associated with ice crystal formation. Preliminary analysis on the signal intensities of CKs between fresh and frozen CAMLs are similar, however, the CAML sample size is not large enough to draw definitive conclusions.

We observed from the matched samples that the freeze/thaw process has no adverse effect on morphologic characteristics and the biomarkers of RCC-associated cells. Although we did not observe statistically significant changes in enumeration of spiked breast and prostate cell lines, further study is required to evaluate whether our cryopreservation process is compatible with other tumor types. Furthermore, during the course of investigation, we also found denucleated CAMLs in frozen samples from a patient with RCC, consistent to fresh samples. The denucleated CAMLs resemble typical CAMLs closely in morphologic characteristics and staining patterns except for a lack of nuclei. However, the importance of these structures is undefined.

## Conclusions

Tumor-associated cells can be recovered successfully from cryopreserved PBMC samples with CellSieve™ microfiltration. The morphologic features, biomarkers, and enumeration of tumor-associated cells are preserved in cryopreserved specimens. Application of CellSieve™ microfiltration for detection of tumor-associated cells in cryopreserved PBMC samples allows the biobanking of samples to be processed in batches, to minimize interassay variations, and also enables large-scale, long-term retrospective and epidemiologic analyses in selected cancer populations.

## Additional files

10.1186/s12967-016-0953-2 Limit of detection (LOD) of 6 representative cell lines. Exact number of cell inputs and exact number of cells remaining on filter were determined as previously described in [1]. Briefly, cell lines were centrifuged at 125×*g* for 10 min and the cell pellet was suspended in 1 mL staining solution, containing 1 × PBS and 5 µM DAPI. Cells were incubated for 20 min, centrifuged at 125×*g* for 10 min and the cell pellet was washed with 1 mL PBS, centrifuged at 125×*g* for 10 min and finally suspended in 200 uL PBS. 1–5 µL of the stained cells were placed on a microscope slide and exact cell counts were obtained using a DAPI fluorescent channel. The microscope slides with stained pre-counted cells were then washed into samples prior to experiment. LOD experiments for each cell line, n = 9.
10.1186/s12967-016-0953-2 Recovery efficiency of spiked HUVECs.
10.1186/s12967-016-0953-2 Isolation and identification of primary human umbilical vein endothelial cells (HUVEC) using CellSieve™ microfiltration system. The filter-captured HUVEC cells were stained with endothelial markers CD31 (green) and CD146 (red). Cell nuclei were counterstained with DAPI (blue). The capture efficiency of HUVEC cells was 96.3 ± 4.0 %, n = 3.
10.1186/s12967-016-0953-2 Effect of storage temperature on peripheral blood mononuclear cell (PBMC) samples. (A) MCF7; (B) 786-O; (C) PBMC. The PBMC cells were prepared from whole blood samples collected in heparin tube from healthy controls by using standard Ficoll protocols. The PBMCs were resuspended in cryofluid to a final concentration of 1 million/mL and then aliquoted to 1 mL per vial in cryotube. Breast cancer cell line, MCF7 and RCC cell line, 786-O were spiked in the PBMC at ratio of 100 tumor cells per million of PBMC, respectively. The spiked PBMC samples (n = 3 for each condition) were stored at −80 °C and liquid nitrogen. After three weeks of storage, the samples were thawed out and processed through standard CellSieve™ microfiltration and antibody staining. No cell damage was observed after storage for three weeks in lower temperature condition in liquid nitrogen. We did not find differences in cell morphology and antibody staining patterns between the cells stored at −80 °C and liquid nitrogen.
10.1186/s12967-016-0953-2 Comparison of signal intensity between fresh and frozen Cancer-Associated Macrophage-like Cells (CAMLs) in RCC patient 1 samples collected at three time points. The cytoplasmic signal of CKs was measured in the fresh CAMLs without cryopreservation and in the frozen CAMLs with cryopreservation for 7 days. The CK signals are plotted versus the CAML cells. The error bars indicate the high and low CK signals in each CAML. The dotted line indicates the background signal of the filter membrane.
10.1186/s12967-016-0953-2 A mitotic CTC in a cell cluster detected in frozen RCC Patient 2 sample. The image is cropped to show the mitotic CTC. The CTC is under cell division (red arrow). The mitotic CTC is seen at telophase because the nuclei have been separated into 2 sets of chromosomes (light blue) and share a common cytoplasm (green).

## Abbreviations

ATCC: American Type Culture Collection
CAMLs: cancer-associated macrophage-like cells
CK: cytokeratin
CTCs: circulating tumor cells
DAPI: 4′,6-diamidino-2-phenylindole
EF615: eFluor^®^ 615
EpCAM: epithelial cell adhesion molecule
FITC: fluorescein
IRB: Institutional Review Board
LN2: liquid nitrogen
PBMCs: peripheral blood mononuclear cells
PBS: phosphate buffered saline
PD-L1: programmed death-ligand 1
PE: phycoerythrin
RCC: renal cell carcinoma
SD: standard deviation
SEM: scanning electron microscope
