# Supplementary material for: Detection of tumor-associated cells in cryopreserved peripheral blood mononuclear cell samples for retrospective analysis
Source: J Transl Med. 2016 Jul 2;14:198. doi: 10.1186/s12967-016-0953-2 (PMC4930561; doi:10.1186/s12967-016-0953-2)
Supplement: Supplementary file 1 — 10.1186/s12967-016-0953-2 Limit of detection (LOD) of 6 representative cell lines. Exact number of cell inputs and exact number of cells remaining on filter were determined as previously described in [1]. Briefly, cell lines were centrifuged at 125×g for 10 min and the cell pellet was suspended in 1 mL staining solution, containing 1 × PBS and 5 µM DAPI. Cells were incubated for 20 min, centrifuged at 125×g for 10 min and the cell pellet was washed with 1 mL PBS, centrifuged at 125×g for 10 min and finally suspended in 200 uL PBS. 1–5 µL of the stained cells were placed on a microscope slide and exact cell counts were obtained using a DAPI fluorescent channel. The microscope slides with stained pre-counted cells were then washed into samples prior to experiment. LOD experiments for each cell line, n = 9. [file 12967_2016_953_MOESM1_ESM.pptx]

## Slide 1
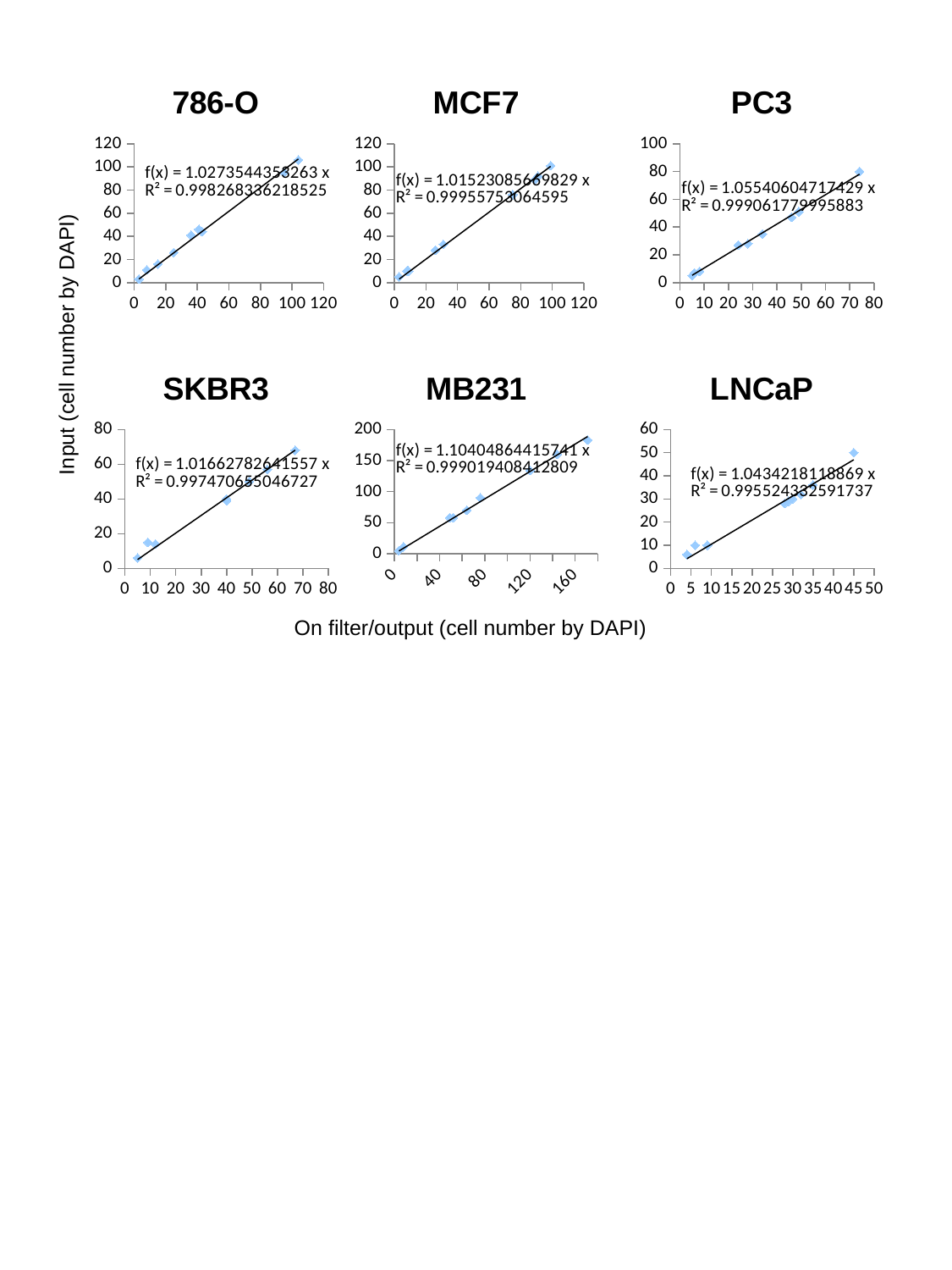

### Chart: 786-O
| Category | |
|---|---|
### Chart: MCF7
| Category | |
|---|---|
### Chart: PC3
| Category | |
|---|---|Input (cell number by DAPI)
### Chart: SKBR3
| Category | |
|---|---|
### Chart: MB231
| Category | |
|---|---|
### Chart: LNCaP
| Category | |
|---|---|On filter/output (cell number by DAPI)
