# Supplementary material for: Detection of tumor-associated cells in cryopreserved peripheral blood mononuclear cell samples for retrospective analysis
Source: J Transl Med. 2016 Jul 2;14:198. doi: 10.1186/s12967-016-0953-2 (PMC4930561; doi:10.1186/s12967-016-0953-2)
Supplement: Supplementary file 2 — 10.1186/s12967-016-0953-2 Recovery efficiency of spiked HUVECs. [file 12967_2016_953_MOESM2_ESM.docx]

|  | Input cells | Cells Recovered, No. | Recovery, % |
| --- | --- | --- | --- |
| HUVEC | 17 | 16 | 94.1 |
| HUVEC | 77 | 72 | 93.5 |
| HUVEC | 128 | 127 | 99.2 |

**Supplementary Table S1.** Recovery efficiency of spiked HUVECs
