# Supplementary figures and images for: Detection of tumor-associated cells in cryopreserved peripheral blood mononuclear cell samples for retrospective analysis
Source: J Transl Med. 2016 Jul 2;14:198. doi: 10.1186/s12967-016-0953-2 (PMC4930561; doi:10.1186/s12967-016-0953-2)

## Slide 1
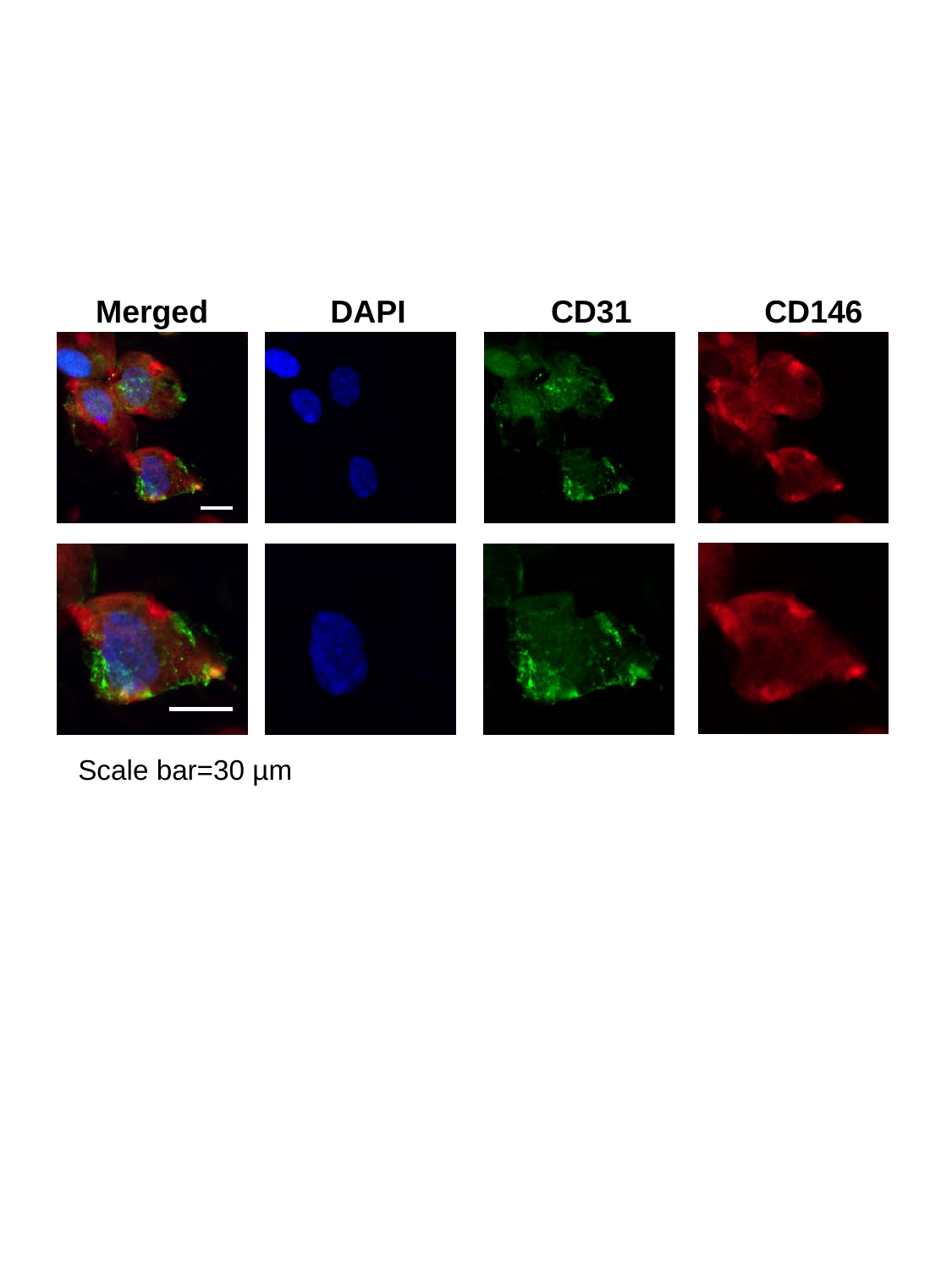

Merged
DAPI
CD31
CD146
Scale bar=30 µm

Supplement: Supplementary file 3 — 10.1186/s12967-016-0953-2 Isolation and identification of primary human umbilical vein endothelial cells (HUVEC) using CellSieve™ microfiltration system. The filter-captured HUVEC cells were stained with endothelial markers CD31 (green) and CD146 (red). Cell nuclei were counterstained with DAPI (blue). The capture efficiency of HUVEC cells was 96.3 ± 4.0 %, n = 3. [file 12967_2016_953_MOESM3_ESM.pptx]

## Slide 1
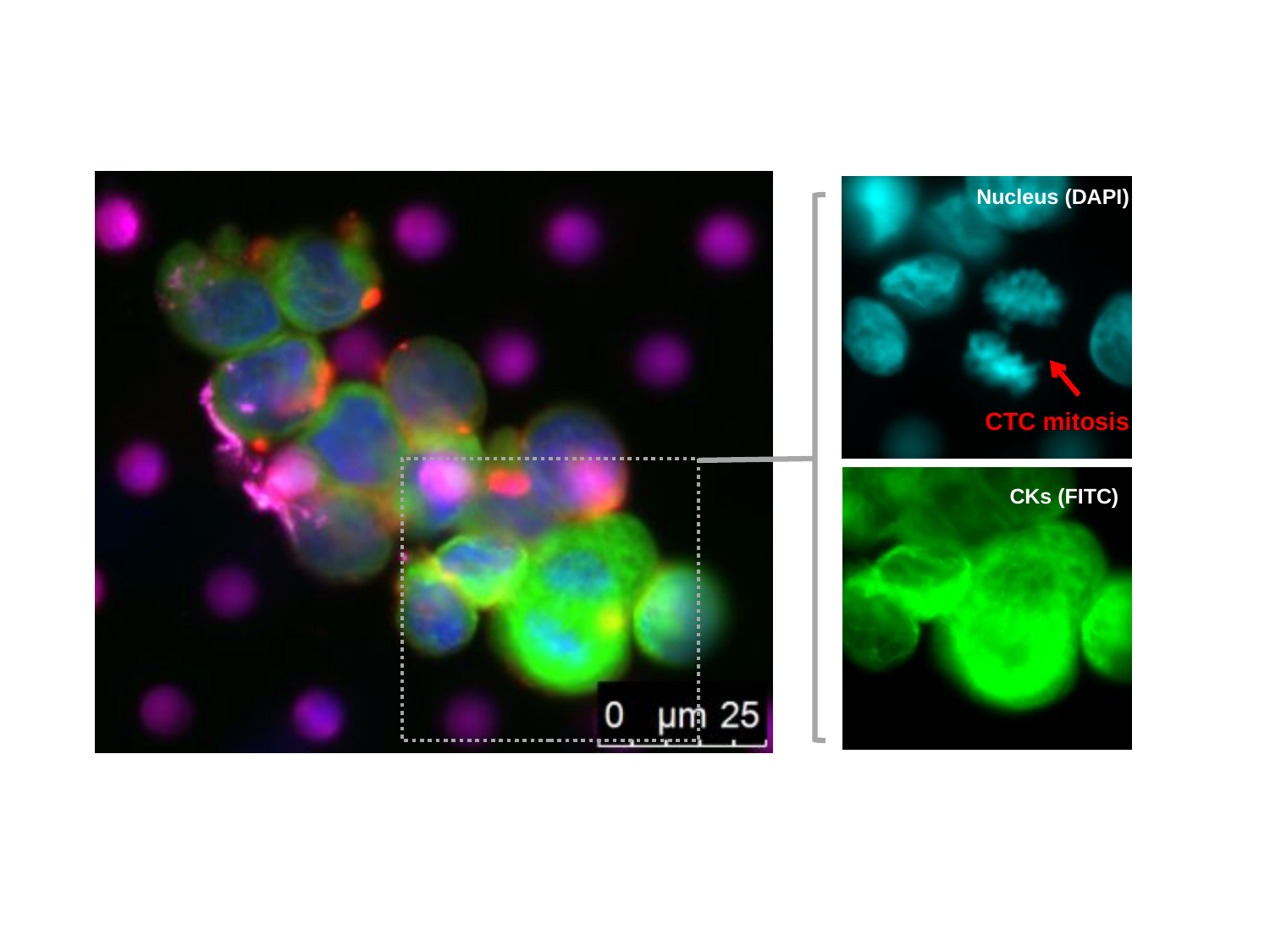

Nucleus (DAPI)
CTC mitosis
CKs (FITC)

Supplement: Supplementary file 6 — 10.1186/s12967-016-0953-2 A mitotic CTC in a cell cluster detected in frozen RCC Patient 2 sample. The image is cropped to show the mitotic CTC. The CTC is under cell division (red arrow). The mitotic CTC is seen at telophase because the nuclei have been separated into 2 sets of chromosomes (light blue) and share a common cytoplasm (green). [file 12967_2016_953_MOESM6_ESM.ppt]
