# Supplementary material for: Detection of tumor-associated cells in cryopreserved peripheral blood mononuclear cell samples for retrospective analysis
Source: J Transl Med. 2016 Jul 2;14:198. doi: 10.1186/s12967-016-0953-2 (PMC4930561; doi:10.1186/s12967-016-0953-2)
Supplement: Supplementary file 4 — 10.1186/s12967-016-0953-2 Effect of storage temperature on peripheral blood mononuclear cell (PBMC) samples. (A) MCF7; (B) 786-O; (C) PBMC. The PBMC cells were prepared from whole blood samples collected in heparin tube from healthy controls by using standard Ficoll protocols. The PBMCs were resuspended in cryofluid to a final concentration of 1 million/mL and then aliquoted to 1 mL per vial in cryotube. Breast cancer cell line, MCF7 and RCC cell line, 786-O were spiked in the PBMC at ratio of 100 tumor cells per million of PBMC, respectively. The spiked PBMC samples (n = 3 for each condition) were stored at −80 °C and liquid nitrogen. After three weeks of storage, the samples were thawed out and processed through standard CellSieve™ microfiltration and antibody staining. No cell damage was observed after storage for three weeks in lower temperature condition in liquid nitrogen. We did not find differences in cell morphology and antibody staining patterns between the cells stored at −80 °C and liquid nitrogen. [file 12967_2016_953_MOESM4_ESM.pptx]

## Slide 1
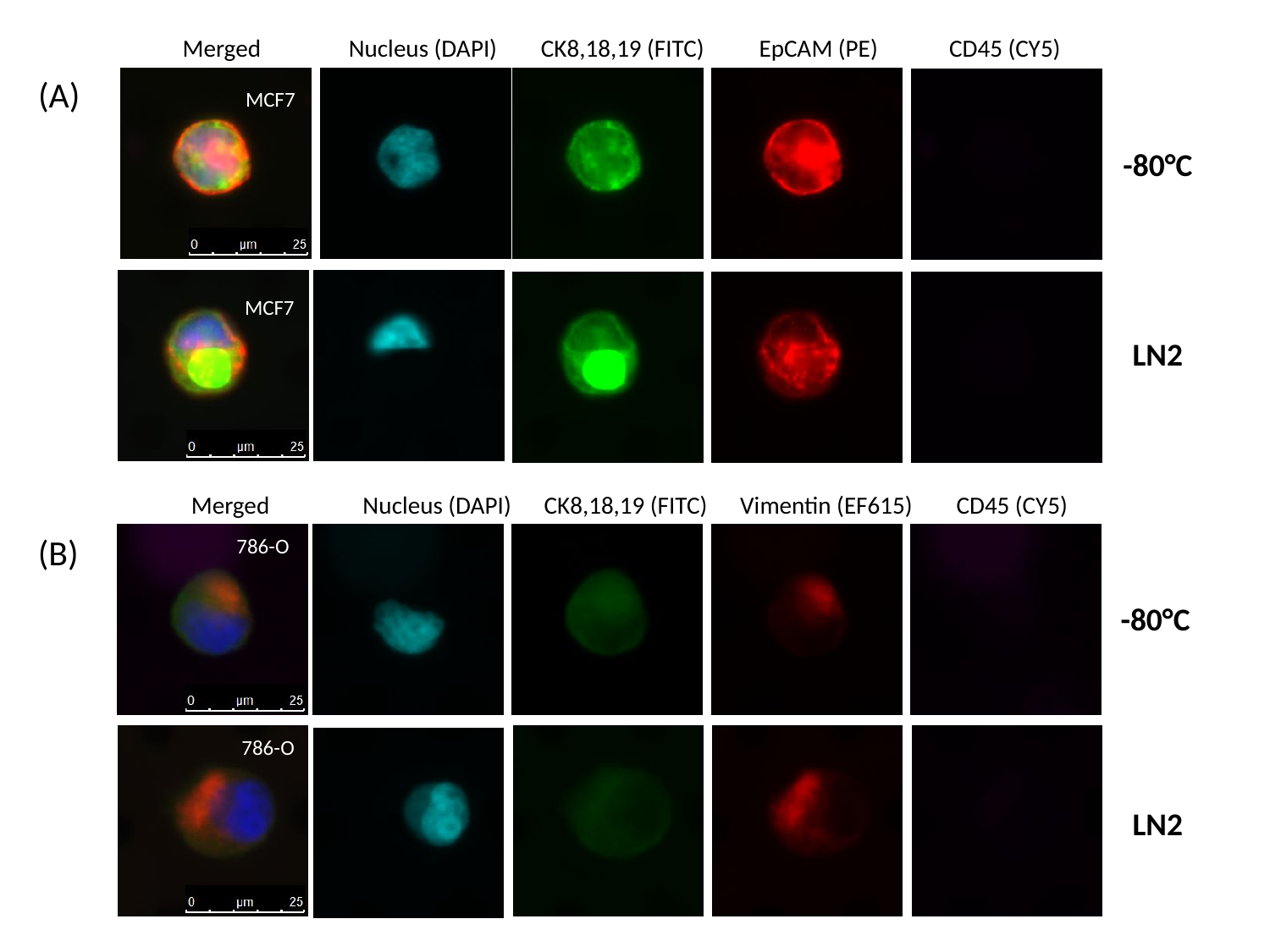

Merged Nucleus (DAPI) CK8,18,19 (FITC) EpCAM (PE) CD45 (CY5)
(A)
MCF7
-80°C
MCF7
LN2
Merged Nucleus (DAPI) CK8,18,19 (FITC) Vimentin (EF615) CD45 (CY5)
(B)
786-O
-80°C
786-O
LN2

## Slide 2
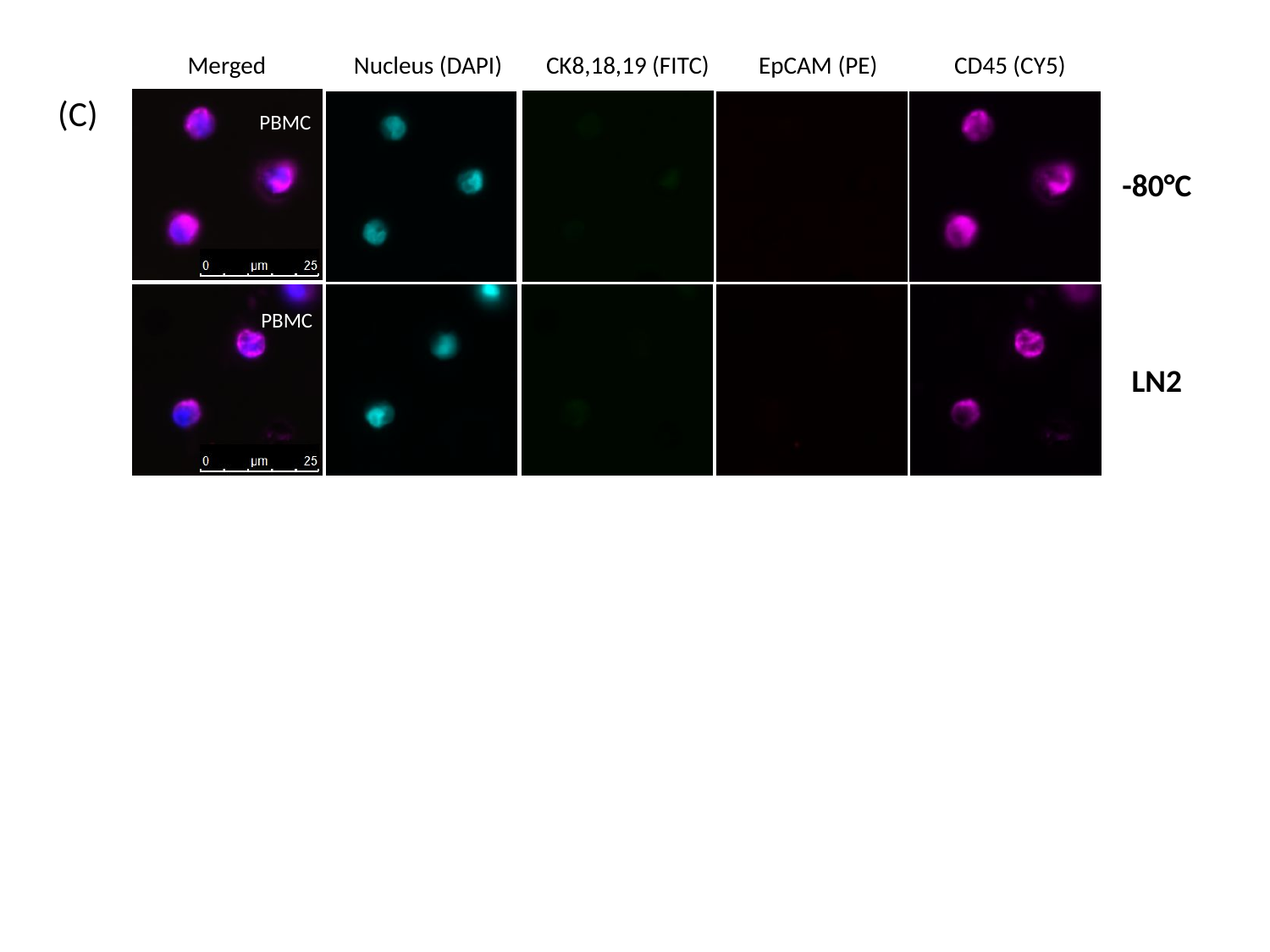

Merged Nucleus (DAPI) CK8,18,19 (FITC) EpCAM (PE) CD45 (CY5)
(C)
PBMC
-80°C
PBMC
LN2
