# Supplementary material for: Detection of tumor-associated cells in cryopreserved peripheral blood mononuclear cell samples for retrospective analysis
Source: J Transl Med. 2016 Jul 2;14:198. doi: 10.1186/s12967-016-0953-2 (PMC4930561; doi:10.1186/s12967-016-0953-2)
Supplement: Supplementary file 5 — 10.1186/s12967-016-0953-2 Comparison of signal intensity between fresh and frozen Cancer-Associated Macrophage-like Cells (CAMLs) in RCC patient 1 samples collected at three time points. The cytoplasmic signal of CKs was measured in the fresh CAMLs without cryopreservation and in the frozen CAMLs with cryopreservation for 7 days. The CK signals are plotted versus the CAML cells. The error bars indicate the high and low CK signals in each CAML. The dotted line indicates the background signal of the filter membrane. [file 12967_2016_953_MOESM5_ESM.ppt]

## Slide 1
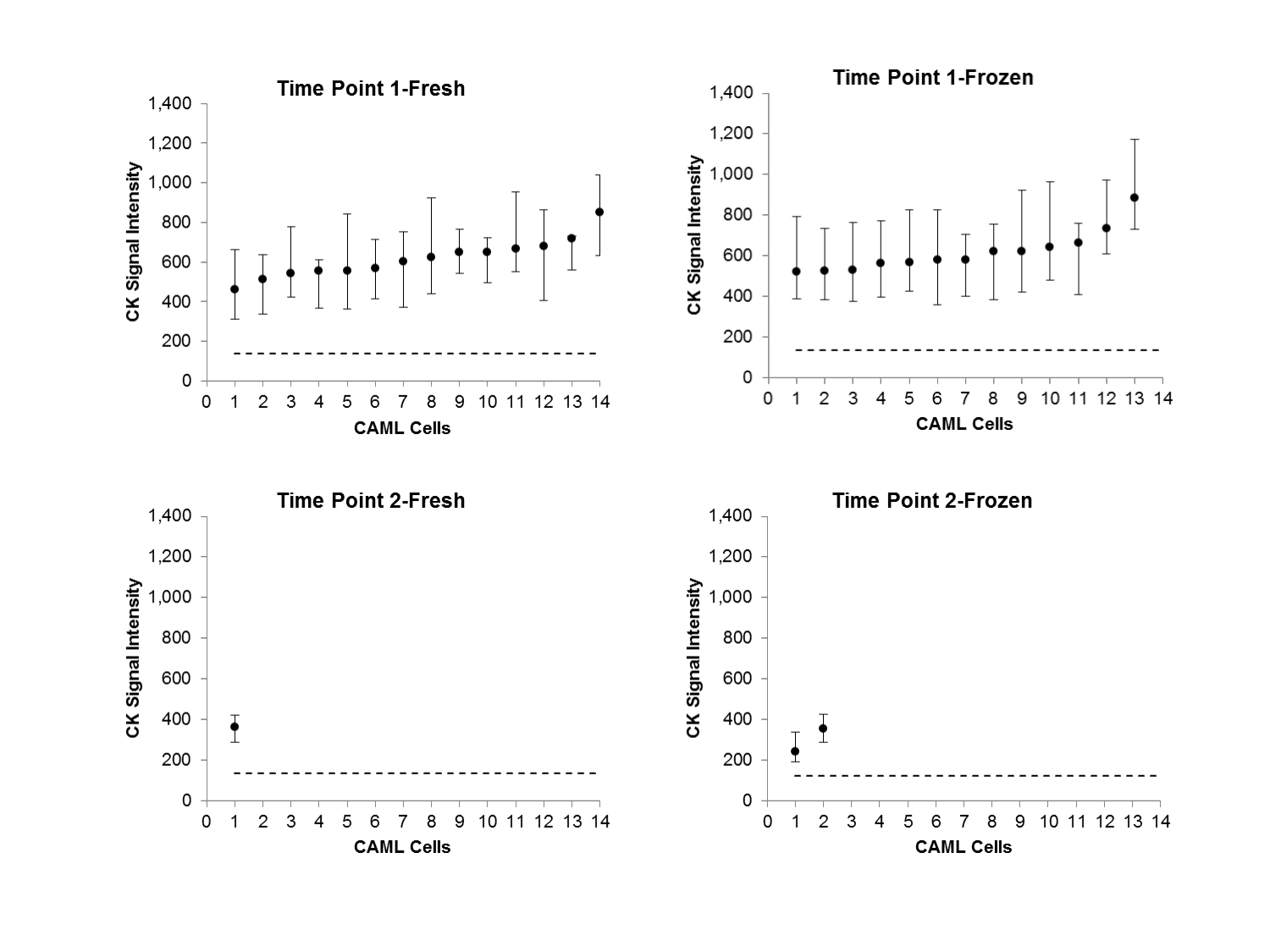

## Slide 2
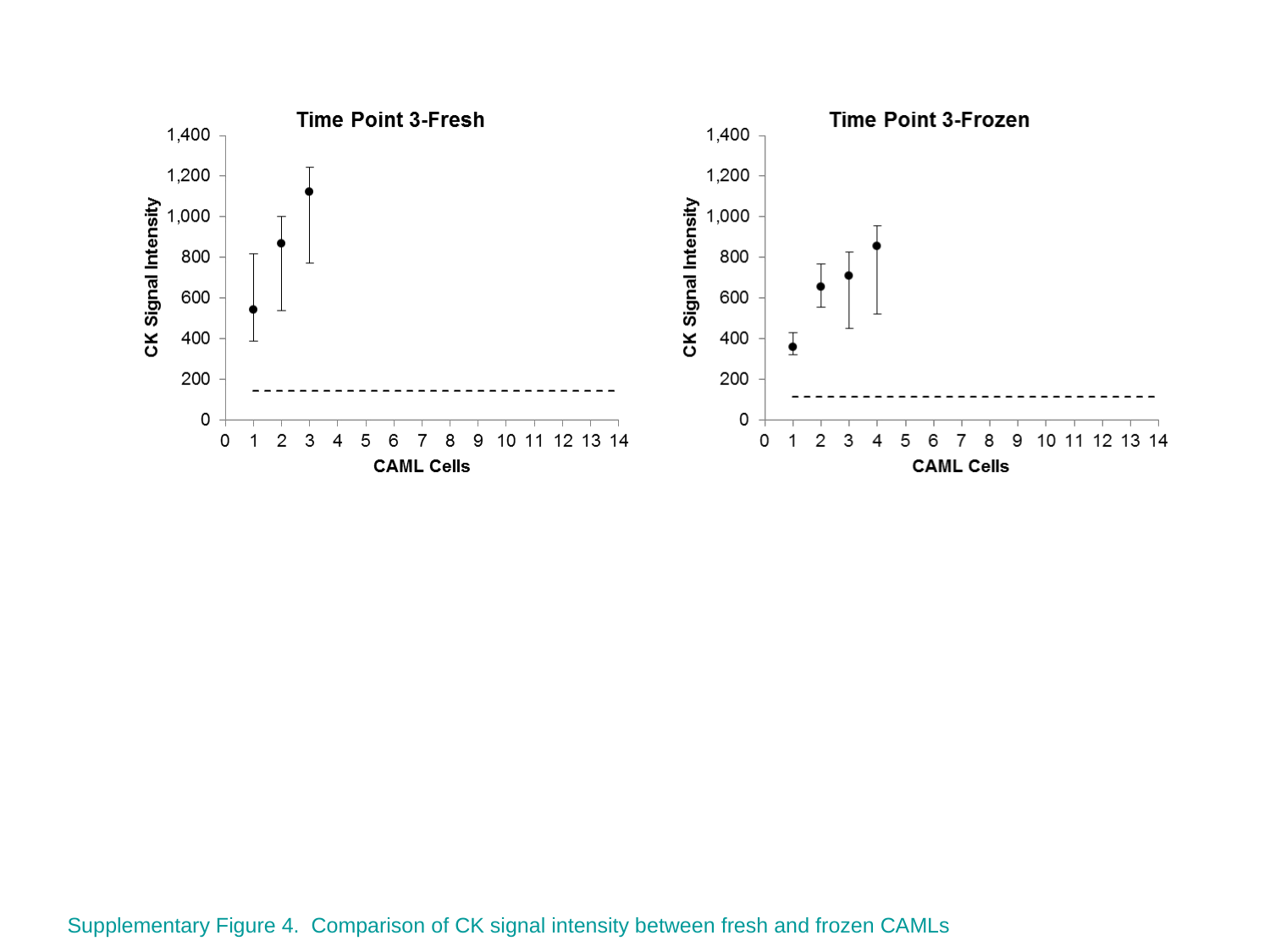

Supplementary Figure 4. Comparison of CK signal intensity between fresh and frozen CAMLs
